# Evidence-based practice in primary healthcare from the managerial point of view – a national survey

**DOI:** 10.1186/s12913-021-07023-w

**Published:** 2021-09-26

**Authors:** Tobias Abelsson, Helena Morténius, Ann-Kristin Karlsson, Stefan Bergman, Amir Baigi

**Affiliations:** 1 Department of Research and Development, Region Halland, Region Halland, R&D, Box 517, 301 80 Halmstad, Sweden; 2 Primary Healthcare Unit, Department of Public Health and Community Medicine, Institute of Medicine, The Sahlgrenska Academy, University of Gothenburg, Gothenburg, Sweden; 3 Department of Healthcare, Region Halland, Halmstad, Sweden; 4 Department of Research and Development, Spenshult, Halmstad, Sweden

**Keywords:** Diffusion of innovation, Evidence-based practice, Evidence-based medicine, Healthcare management, Information-seeking behaviour, Knowledge management, Primary healthcare

## Abstract

**Background:**

The vast availability of and demand for evidence in modern primary healthcare force clinical decisions to be made based on condensed evidence in the form of policies and guidelines. Primary healthcare managers play a key role in implementing these governing documents. Thus, the aim of this article is to investigate the use and availability of evidence-based practice resources from the perspective of first-line primary healthcare managers.

**Methods:**

The study is based on a national survey of primary healthcare managers, consisting of 186 respondents, recruited nationally from Sweden. The data was analysed using empirically constructed concepts and validated using factor analysis. A chi-square test was utilized to determine the statistical significance of comparisons. Associations between variables were calculated using Spearman’s correlation coefficients. All tests were two-sided, and the significance level was set to 0.05.

**Results:**

A majority (97 %) of managers stated that guidelines and policy documents impacted primary healthcare; 84 % of managers observed a direct effect on daily practices. Most of the managers (70 %) stated that some adaptation was needed when new evidence was introduced. The managers emphasized the importance of keeping themselves updated and open to new information about work routines (96 %).

**Conclusions:**

The study illustrates a nearly unanimous response about the influence of clinical evidence on daily practice. The emphasis on the importance of all staff members keeping their professional knowledge up to date is viewed as a direct result of this effect on daily practice. An information-dense organization such as a primary healthcare organization would have much to gain from increased cooperation with regional information resources such as clinical libraries.

**Supplementary Information:**

The online version contains supplementary material available at 10.1186/s12913-021-07023-w.

## Background

In Sweden, the population has access to tax-funded health and medical care. The foundation of the care system is the county councils, which funds and administrates the regional healthcare [1]. Primary healthcare is provided by county councils and includes overall care and treatment that do not require specialist services. Primary healthcare is provided by minor units: primary healthcare centres (PHCs). The composition of staff and services may vary due to the size of the PHC. However, the least common denominator is that each PHC employs a number of clinical health professionals, such as general practitioners, registered nurses, physiotherapists and social workers specializing in medicine and healthcare.

PHCs are managed by a first-line primary healthcare manager (PHM). The responsibilities may include tasks related to patient safety, coordination and quality of care on a strategic level, economic planning and staffing responsibility [2]. Ultimately, each county council is responsible for precisely defining the duties of the managerial role [2]. In Sweden, official education for PHM is not available, and a manager is usually recruited from experienced co-workers with a clinical background.

One of the key strategic tasks of the PHM is to facilitate meeting the information needs of the staff to ensure that they are able to keep up to date in their profession, and meet the demands of patient-centred care as stated by Swedish law [3, 4].

Patient-centred care rests upon one of the fundamental concepts in modern medicine: All decisions regarding a patient’s health should be made with consideration of the patient’s preferences [5]. Additionally, the best available evidence (e.g., guidelines, study results, reviews, trials, etc.) should be used in conjunction with the knowledge and experience of health professionals (HPs), which is the guiding principle of evidence-based practice (EBP) [6, 7].

EBP challenges both the individual HP and the PHM, as the PHM has a role in facilitating the use of EBP by HPs by ensuring access to information resources and sufficient time in daily practice for HPs to remain current in their professional roles and development [8–12].

The cumulative expectations of HPs regarding availability, information knowhow and communication skills in turn place high demands upon the internal structures of the PHC. All of which depend upon the PHM and her/his management of the PHC to some extent [13–16].

Being but one part of successful EBP implementation, information supply and information retrieval must be considered vital in today’s information-laden primary care. With this said, PHMs tasks are not limited to assuring a successful information supply to the HPs. The PHMs play an important role through the entire implementation process by taking varying roles in different stages of the implementation [17]. A large amount of evidence-based implementation research in healthcare focusing on wider organizational perspectives, such as communication and implementation strategies, has been published [12, 15, 18–24]. Although healthcare managers are frequently mentioned as having key roles in the implementation process [15, 16, 21, 25–29], knowledge of the macro level of the nature of their influence on the circumstances of implementing EBP appears to be lacking.

Some exceptions have been noted; for example, in 2003, a Scottish study investigated the attitudes of six professional groups within primary healthcare and their perspectives on EBP that included PHMs [30]. A systematic review of managers’ influence concerning research use in nursing was also published [21]. The impacts of the personality and professional knowledge of PHMs are important factors contributing to how they choose to lead and influence the rest of the staff, especially in regard to conveying attitudes and encouragement towards the ability of individual HPs to keep up to date and implement EBP [21, 26, 27, 30].

In the context of supplying quality information and the value of assistance in identifying relevant resources, access to clinical library resources (databases, journals, decision support systems, etc.) and information specialists might help HPs by facilitating information exchange and increasing efficiency in information seeking [16, 19, 31]. Information resources are considered vital for a knowledge organization, such as primary healthcare, but might not always be connected to the library concept in the minds of PHMs/HPs, especially if a geographical distance exists between a library and its users [16].

To our knowledge, little or no research has been conducted on the perspectives of first-line primary healthcare managers regarding their habits of keeping up to date professionally and evaluating new information in their occupational role, habits that might have an influence on the facilitation of co-workers’ mandatory roles as up-to-date experts.

The aim of this study was to investigate the availability and employment of evidence-based practice in Swedish primary healthcare facilities from the perspective of first-line primary healthcare managers.

## Methods

The study was designed as a quantitative national online survey based on a questionnaire directed to PHMs holding a managerial position at a public PHC. It focused on their experience evaluating evidence and implementing evidence-based practice.

### Recruitment and study population

The strategic recruitment of respondents was conducted during the first half of 2018. Two inclusion criteria were used: being able to read and write in Swedish and being an employee in a managerial position at a public PHC.

Since a complete national record of PHCs and their staff was unavailable, the research team had to compile this record from an acquired list of the official websites of each county council. The list was acquired from the Swedish Association of Local Authorities and Regions. Guided by this list, the research team then visited each county’s webpage for primary healthcare and manually extracted the names and contact information of the PHM for each individual PHC. These individuals were the recipients of the study introduction letter and questionnaire.

One week after the first invitation letter was sent, a reminder letter was sent to all recipients who had not completed the survey, or had started the survey but not completed it. This process was then repeated two more times at one-week intervals such that the respondents were reminded of the survey a total of four times. Each reminder targeted the non-complete or non-started respondents.

### Power of the study

We did not find any similar study that could serve as a frame of reference with a measurable effect size. Therefore, we decided to invite all primary healthcare managers in the country to participate in our study. Hopefully, the results of this study will generate future hypotheses and thereby contribute to the funding of subsequent studies to determine preliminary effect sizes.

### Questionnaire design and construction

Based on the purpose of the study, a questionnaire consisting of 24 items was constructed. The questionnaire was created based on a literature review and the experience of the research team. It originated from the idea of five question clusters covering the background data (age, education level and the size of the primary healthcare area), evidence-based practice, information retrieval, implementation of new information and knowledge of regional informational service suppliers. The original questionnaire was constructed in Swedish and was deployed as an online questionnaire. A complete English translation of the full questionnaire can be found in supplement 1.

### Analysis

The question clusters were deconstructed during the analysis phase and resulted in the empirical concepts of the study. In order to describe the base influence of information retrieval and evaluation in EBP, a descriptive analysis was then made using factor analysis. The choice of factor analysis was made in order to index activities connected to library operations in a coherent context. The purpose of analysing the background variables where to adjust the possible influence of discrepancies that could influence the internal relationship between the variables.

### Background variables

Education and size of population were together with age and sex of participants used as background variables. The variable “education” divided into “long” (second cycle education and higher) and “short” (first cycle education or below) was added to compare the effect of individual educational attainment on the results. In the same manner, the demographic value of “population” denoted the number of inhabitants in the region where the PHC was located.

### Empirical concepts

Based on the research team’s pre-existing understanding of the research topic, the data were initially sorted into four empirical concepts. A fifth concept, “biolibrary activity”, emerged as a result of the performed factor analysis due to its strong association with library activities. Data from all concepts were operationalized into two subgroups, “education” and “population”, which were subjected to statistical tests to determine their power values.

### Policy and guidelines

This concept encompassed items concerning the application of policy and guideline documents. The concept described the experienced impact of using such policy documents in the clinical setting and the influence of these documents on clinical decisions.

### Capture

The common denominators for the items in this concept were the evaluation and adaptation of evidence intended for use in daily clinical practice. Opinions about the process were investigated to procure and assign value to the information presented to the managers in any form concerning their professional role.

### Access

The attitude towards the importance of occupational information research and keeping up to date were the focus of this concept. The goal was to encompass the experience of finding information and keeping up to date.

### Management

The focus of this concept was the manager’s willingness to change her or his workplace. The questionnaire investigated the manager’s attitude towards implementing change in her or his PHC.

### Library operations

This concept included items concerning the manager’s awareness of library services and their utilization to increase information capture and strengthen the information skills needed to practice EBP.

### Ethics

Participation was voluntary, and confidentiality was guaranteed. The participants were informed about the aim and structure of the study. The study conformed to the principles outlined in the Declaration of Helsinki [32].

The enquiry form was created to be anonymous and only show the results from fully completed forms.

### Statistics

The factor analysis was performed using the protocol reported by Williams et al. [33]. The model provides general guidelines of a minimum sample size of at least 100 participants [33, 34]. This recommendation was met by our sample size, which consisted of 186 complete responses. The Kaiser-Meyer-Olkin (KMO) test and Bartlett’s test of sphericity (BTS) were used to assess the relevance of the model [33, 35, 36]. The data were extracted using principal component analysis [33, 37, 38]. Eigenvalues were set to 1, and the varimax rotation method was chosen. All concepts were created empirically, except for the concept of “biolibrary activity”. These factors were identified during the factor analysis as the single pure factor based on 5 items assessing different aspects of library activities and were named “biolibrary activity” (Table 1).

**Table 1:** The results of the factor analysis of items belonging to biolibrary activity (*n* = 186)

	Impact (%)	Cumulative variance	Cronbach’s alpha^a^
Q12: Current cooperation with clinical library.	38		
Q13: Own knowledge about clinical library services.	54		
Q14: Last contact with the clinical library.	22		
Q15: Continuity of clinical library information.	28		
Q16: Flexibility in reaching clinical library.	54		
BIOLIBRARY ACTIVITY		62.15	0.78

KMO of sampling adequacy: 0.76

Bartlett’s test of sphericity: *p* < 0.0001

^a^Cronbach’s alpha coefficient

A chi-square test was used to compare proportions of items within the four empirical concepts and to determine the statistical significance. Spearman’s correlation coefficients were calculated to examine the statistical correlations of the managers’ attitudes and the items in each concept. All tests were two-sided, and the significance level was set to 0.05

## Results

The final sample consisted of 186 respondents to the 564 requests sent, for a response rate of approximately 33 %. The age of the participants was 30 to 65 years, with a median age of 53 years and a predominance of females (Table 2). The level of education was distributed almost equally, but a small majority had less education than their colleagues (first cycle of education or below). The majority of respondents managed a PHC in a region with less than or equal to 500,000 inhabitants (Table 2).

**Table 2:** Descriptive statistics on the background variables of the population (*n* = 186)

	Observations (n)	Percent (%)
Age		
≤ 53 years	96	51.6
> 53 years	90	48.4
Sex		
Male	30	16.1
Female	156	83.9
Education level		
Short	90	57.7
Long	66	42.3
Population density		
≤ 500 000	96	57.8
> 500 000	70	42.2

Short education = first cycle of education or below

Long education = second cycle of education and higher.

### Policy and guidelines concept

Policy and guidelines exerted a fundamental effect on the primary healthcare organization, according to 97 % of the respondents. This impact was also observed in daily practice, according to 84 % of respondents (Table 3 A).

**Table 3:** Descriptive statistics for the items and concepts included in the study (n=186). The chi square test was utilized to compare each item

		Percent (%)	*p*
POLICY AND GUIDELINES			
Q1: Degree of impact of policy and guidelines on the organization.	Large	97.0	
	Small	3.0	
			<0.0001
Q2: Impact of policy and guidelines on daily practice.	Impact	84.0	
	No impact	16.0	
			<0.001
Q3: Own knowledge of where to find alternate decision support systems.	Yes	70.0	
	No	30.0	
			<0.001
Q4: Co-workers’ knowledge of where to find alternate decision support systems.	Yes	52.0	
	No	48.0	
			0.441
CAPTURE			
Q7: Time spent on reworking or creating policy documents in order to unify practice.	Little	36.0	
	Much	64.0	
			<0.001
Q8: Demands on evidence-based practice that impede on clinical practice.	Rarely	68.0	
	Often	32.0	
			<0.001
Q9: Evaluation of evidence when implementing new treatments.	Yes	75.0	
	No	25.0	
			<0.001
ACCESS			
Q10: Co-workers’ possibilities to research information in order to keep up to date.	Important	96.0	
	Not important	4.0	
			<0.001
Q11: Time spent on researching information about one’s own occupational topics.	≤1 hour/week	15.0	
	2-3 hours/week	70.0	
	>3 hours/week	15.0	
			<0.001
MANAGEMENT			
Q5: Primary healthcare would gain based on faster adaptation to new evidence.	Yes	70.0	
	No	30.0	
			<0.001
Q6: New thoughts and ideas about the work routine are stimulating.	Yes	96.0	
	No	4.0	
			<0.001

Concerning sites to find alternate information about policies and guidelines, 70 % of the responding managers stated that they knew where to find relevant alternative information. They estimated that their co-workers had less knowledge of this sort, but 52 % believed their co-workers possessed this knowledge (Table 3). These answers were all independent of background variables, such as primary healthcare area and level of education (Table 4).

**Table 4:** Descriptive statistics on the items and concepts divided by education level and population size (*n*=186)

		Education			Population		
		Short *N*=90	Long *N*=66		<500 000 *N*=80	≥500 000 *N*=57	
		Percent (%)		*p*	Percent (%)		p
POLICY AND GUIDELINES							
Q1: Degree of impact of policy and guidelines on the organization	Large	96.0	99.0		96.0	99.0	
				0.215			0.243
Q2: Impact of policies and guidelines on daily practice	Evident impact	80.0	86.4		76.1	82.8	
				0.580			0.305
Q3: Own knowledge of where to find alternate decision support systems	Yes	72.0	70		73.0	71.0	
				0.786			0.777
Q4: Co-workers’ knowledge of where to find alternate decision support systems	Yes	72.0	72.0		70.0	76.0	
				0.999			0.394
CAPTURE							
Q7: Time spent on reworking or creating policy documents in order to unify practice.	Little	33.0	39.0		33.0	37.0	
				0.441			0.594
Q8: Demands on evidence-based practice that impede on clinical practice.	Rarely	74.0	59.0		77.0	81.0	
				0.049			0.536
Q9: Evaluation of evidence when implementing new treatments	Yes	74.0	80.0		76.0	67.0	
				0.384			0.203
ACCESS							
Q10: Co-workers’ possibilities to research information in order to keep up to date.	Important	94.0	95.0		94.0	97.0	
				0.789			0.370
Q11: Time spent on researching information about one’s own occupational topics.	≤1 hour/week	8.0	21.0		13.0	13.0	
	2-3 hours/week	79.0	61.0		74.0	71.0	
	>3 hours/week	13.0	18.0		13.0	16.0	
				0.054			0.912
MANAGEMENT							
Q5: Primary healthcare would gain based on faster adaptation to new evidence	Yes	70.0	74.0		68.8	74.3	
				0.585			0.618
Q6: New thoughts and ideas about the work routine are stimulating.	Yes	98.0	97.0		97.0	98.0	

The exception was the item “Possibilities for co-workers to research information in order to keep up to date.” The item describes a significant difference in the view of EBP as being a burden in daily practice. The distribution of answers showed that respondents with shorter education tended to view the demands of EBP as more burdensome than colleagues with longer education (Table 4)

### Capture concept

A majority (70 %) of the managers in this study answered that some time was needed to adapt new evidence before implementing it in practice. 68 % stated that clinical practice was rarely inhibited by the demands of being evidence-based (Table 3).

All items except one in this concept were independent of background variables such as the managers’ primary healthcare area and educational level (Table 4), except for the item that addressed managers’ educational level. Managers with longer education found the demand for evidence in practice less of an obstruction than their colleagues with shorter education (Table 4).

### Access concept

Almost all (96 %) of the managers understood the importance of their staff keeping up to date on an individual level. The respondents generally stated that they dedicated up to three hours per week to remain up to date in their profession. Some differences were observed based on the primary healthcare area and the managers’ allocated time for their own research. Namely, managers in larger areas expended more time in personal occupational research (Table 4). No other differences were observed based on the background variables.

### Management concept

96 % of the managers had a positive attitude towards new ideas and thoughts about work routines. A majority of the respondents (70 %) also thought that primary healthcare in general would benefit from a faster adaptation of new evidence (Table 3). As in the previous concept, the results were independent of the two background variables (Table 4).

### Library operations concept

Focusing on managers’ knowledge of and/or cooperation with the regional clinical library, the items in this concept encompassed the managers’ knowledge of library resources. This concept was most actualised in cases when managers stated the importance of EBP in combination with knowledge of clinical library resources. As a concept characterised by many coherent items, library operations was the only concept that was able to be validated using factor analysis (Table 2).

### The factor of biolibrary activity

A factor analysis was performed to verify the level of covariance in items concerning library operations displayed in Table 1. The results of the Kaiser-Meyer-Olkin test and Bartlett’s test of sphericity were KMO = 0.78 and BTS = 245.6; *p* < 0.0001. The results of the factor analysis revealed one pure factor, “biolibrary activity”, with 62.15 degrees of cumulative variance. Cronbach’s alpha coefficient indicating the degree of internal consistency was within the recommended range.

### Association between management’s positive attitude and EBP

Management’s positive attitude through the adaptation of new evidence and new thoughts was revealed in the correlation between Q11 “New thoughts and ideas about the work routine are stimulating” and items Q1 “Degree of policy/guideline impact on the organization” (r = 0.48; *p* < 0.001) and Q6 “Demands on evidence-based practice impede clinical practice” (r = 0.23; p = 0.031). The positive attitude of management regarding new routines and thoughts in the primary healthcare context was illustrated by the correlation between Q10 “Primary healthcare would gain based on faster adaptation to new evidence” and items Q1 “Degree of policy/guideline impact on the organization” (r = 0.48; *p* < 0.001), Q6 “Demands on evidence-based practice impede clinical practice.” (r = 0.23; p = 0.031) and Q4 “Co-workers knows where to find alternate decision support systems” (r = 0.21; p < 0.017). These items were, for example, the degree to which the impacts of policies and guidelines on the organization positively correlated with co-workers’ increased opportunities for obtaining up-to-date information within the organization. Consistently, the management’s promotional attitude was positively and significantly correlated with all three concepts: policy and guidelines, capture and access.

## Discussion

The main result was that almost all survey respondents were impacted by policies and guidelines in clinical daily practice. Almost all respondents were positive about change and new ideas regarding the implementation of EBP. According to previous studies, this type of positive thinking among managers might not represent the thoughts among a majority HPs within a changing organization. A certain level of reservation and scepticism regarding the introduction of new guidelines is common [9, 39].

More than half of the PHMs indicated a perceived need to change guidelines or policies for better integration into daily practice. The prerequisite to be able to complete this task would, amongst other things, be to possess sufficient information retrieval skills. According to the literature, information-seeking skills are one of the more common challenges for PHMs [19, 28, 40, 41]. However, the PHMs who answered our questionnaire generally responded that they spent a mean of 2–3 h/week researching or keeping up to date within their profession. This fact might indicate a widespread habit of navigating information resources and knowledge of evaluating research, thus the PHM would not need to spend an excessive amount of time fetching information to keep up to date.

Alternatively, and most likely, the time spent and stated knowledge level in information retrieval might result from restrictive factors such as organizational demands on time management [19, 28, 39, 42]. The literature states that some major barriers to information retrieval by HPs are related to resources such as time allocation towards research [18, 19, 28, 41].

PHMs had some knowledge of the existence of the clinical library. However, library services were rarely used or marketed in such a way that the PHMs understood a practical adaptation of the skillsets and resources offered by the librarians. The indication that some PHMs neither knew about nor used the available library resources is troublesome. Problems related to information requisition and interpretation have been associated with knowledge gaps in previous studies [16, 19, 28, 31, 41].

Therefore, greater cooperation between PHCs and regional clinical libraries would be beneficial. In addition to helping the individual manager information retrieval skills, the efficiency of information retrieval is increased [16, 43]. Clinical library resources have additional benefits such as increased efficiency in information dissemination, assistance by providing alternate examples on a research topic (diffusion of innovation), increased information skills among staff and, most obviously, the provision of trusted information resources. These resources would provide a long-term benefit in terms of the application of EBP and ultimately patient safety [16, 43].

Still, the information resources and its use, albeit criticallyimportant, are only one aspect of a successful implementation strategy since a manager’s job does not solely consist of supplying the HPs with information resources and time to study them. As mentioned earlier the leadership, organizational facilitation and ability to encourage co-workers to implement a percieved strenuous modus of work in order to amplify an effective EBP is also important [17, 21]. However, this fall outside of the scope of this study.

According to our results, respondents with longer education appeared to consider the absolute demand of evidence as less of an impediment in daily practice more frequently than colleagues with a shorter education. This finding is interesting since our results describe a difference in the perception of evidence and meaning of EBP in terms of efforts among respondents depending on their educational background. This phenomenon is also supported by an earlier study showing that managers’ attitudes towards research might result from their own education level and appreciation of EBP [30].

Indeed, there is research that investigates the information seeking behaviours of HPs that shows that they assign different value to different information resources depending on it being physical textbooks or electronically available research articles [44]. Interestingly enough this Japanese study identified a difference between nurses more often choosing printed resources and doctors preferring electronic ones. This could further strengthen our argument that education level may play a role when valuing information resources. It is not implausible to think that these kind of values follows a PHM that have been promoted from the PHC staff.

The aim of this study was to investigate managers’ experiences of working according to the principles of EBP. Judging from the results, managers’ attitudes towards evidence and implementing change generally appear to be positive. This finding is important since managers’ practices of assigning priority and positive values might have a direct influence on the local work environment in either direction [21].

A final reflection upon the positive attitude towards guidelines and policies might be that primary healthcare is governed by laws that incorporate the principles of EBP. By implication, managers should be aware of the concept of evidence by default and should do anything in their power to encourage EBP in the workplace and encourage their individual co-workers to strive to keep themselves updated. But this is a topic that needs further research.

### Limitations of the study

In the manual collection of contacts, a probability of mistyping or receiving dated or even wrong contact information is always present. The request to participate being ignored by recipients was also prominent.

The sample included all available participants, and the total sample size was considered good based on the general consensus in the literature [30]. However, despite four reminders, the response rate of 33 % potentially reduced the strength of the study. It might also introduce bias, as managers interested in the subject might be more prone to answer.

The study is built upon self-reported data, which could be strengthened by future studies that corroborate managers’ information-seeking behaviours.

The study focuses upon limited part of the implementation process and does not offer a complete depiction of the entire process to incorporate new knowledge. It does however contribute to the understanding of the PHMs assigned value to information resources and its availability.

## Conclusions

The main results of the survey indicate a strong managerial awareness of EBP and its importance to primary healthcare. The heavy emphasis upon evidence and its application often leads to a need for adaptation of the material to fit daily practice. Almost all managers underlined the importance of all HPs, including themselves, keeping up to date. The efficiency of this knowledge upkeep might be increased if staff allocated more resources related to information retrieval and information-seeking behaviour, which are services that are offered by local clinical libraries.

## Supplementary Information

**Additional file 1:**  Questionnaire. The translated version of the original Swedish questionnaire.

## Data Availability

The data that support the findings of this study are available from Region Halland. Data were analysed by employees of Region Halland. Restrictions apply to the availability of these data, which were used under licence for the current study and thus are not publicly available. Data are, however, available from the authors upon reasonable request and with permission of Region Halland.

## Abbreviations

BTS: Bartlett’s test of sphericity
EBP: Evidence-based practice
HP: Health professional
KMO: Kaiser-Meyer-Olkin
PHC: Primary healthcare centre
PHM: Primary healthcare manager
