# Supplementary material for: Evidence-based practice in primary healthcare from the managerial point of view – a national survey
Source: BMC Health Serv Res. 2021 Sep 26;21:1014. doi: 10.1186/s12913-021-07023-w (PMC8474930; doi:10.1186/s12913-021-07023-w)
Supplement: Supplementary file 1 — Additional file 1: Questionnaire. The translated version of the original Swedish questionnaire. [file 12913_2021_7023_MOESM1_ESM.docx]

Questionnaire

Section 1: Background
The first 5 queries are about your background and the professional role.

1: Sex

- Woman
- Man
- Other/don’t want to answer.

2: Age:

3: Which is your professional title?

4: Population of your county/region.
(Please mark the alternative below which best corresponds to the size of the region or county you are working in)

- Less than 500.000 inhabitants
- 500.000 to 1.000.000 inhabitants
- More than 1.000.000 inhabitants
- Don’t know.

5: Highest completed academic degree:

- Second cycle education
- Third cycle education
- Other

Section 2: Evidence at the workplace
The following 5 questions are meant to explore how you and your organization implement evidence and evidence based practice.

6: How much time and effort does your organization put in to working with standards and policy documents? (Please mark your answer in the scale 1-5 due to your experience)

- 1 (Little time)
- 2
- 3
- 4
- 5 (Much time)

7: To what extent do you think policy documents such as PM, and standards have an evident influence in daily practice? (Please mark your answer in the scale 1-5 due to your experience)

- 1 (Small)
- 2
- 3
- 4
- 5 (Large)

8: In your workplace; how much time and effort are put upon reworking standards and policy documents in order to unify daily practice such as diagnosis and treatment?
(Please mark your answer in the scale 1-5 due to your experience)

- 1 (Little time)
- 2
- 3
- 4
- 5 (Much time)

9: Do you experience hindrance in daily practice due to the demand of working according to evidence based practice? (Please mark your answer in the scale 1-5 due to your experience)

- 1 (Seldom)
- 2
- 3
- 4
- 5 (Often)

10: Does your organization evaluate new evidence at the managerial level before implementing new practices?

- Yes
- No

Section 3: Information retrieval
The following 4 questions concerns your informational habits and knowledge of where to find information when it's needed.

11: As a manager, how important it is for you that your employees practice individual information retrieval, in order to develop professional knowledge and a high quality care?

- 1 (Not important)
- 2
- 3
- 4
- 5 (Important)

12: How often do you search for research information related to your occupational role?
(Please estimate the approximate time per week you spent on information retrieval, during a typical week.)

- Less than 1 hour/week
- 1-2 hours/week
- 3-4 hours/week
- 5 hours or more/week

13: Do you know where to find alternate decision support systems when ordinary standards or policy documents don´t suffice? (Please mark your answer in the scale 1-5 due to your experience)

- 1 (I don’t know where to find alternate information)
- 2
- 3
- 4
- 5 (I know where to find alternate information)

14: Do you think your employees know where to find alternate decision support systems when ordinary standards or policy documents don´t suffice? (Please mark your answer in the scale 1-5 due to your experience)

- 1 (They don’t know where to find alternate information)
- 2
- 3
- 4
- 5 (They know where to find alternate information)

Section 4: To work with new information
The following 4 questions concerns your organizations way to process and implement new ways of practice and ideas.

15: Do you agree that the primary health care would gain in faster adaptation to new evidence? (Please mark your answer in the scale 1-5 due to your experience)

- 1 (I don’t agree)
- 2
- 3
- 4
- 5 (I agree)

16: In what extent do you view new thought and ideas concerning daily practice as stimulating? (Please mark your answer in the scale 1-5 due to your experience)

- 1 (Small extent)
- 2
- 3
- 4
- 5 (Large extent)

17: In what extent do you view new thought and ideas concerning the daily practice as burdening? (Please mark your answer in the scale 1-5 due to your experience)

- 1 (Small extent)
- 2
- 3
- 4
- 5 (Large extent)

18: How difficult is it to implement new practices in your organization?

- 1 (Difficult)
- 2
- 3
- 4
- 5 (Not difficult)

Section 5: Relationship between the primary health care and the regional informational service suppliers.
The last 5 questions address to the local cooperation between the primary health care centres and the regional informational service suppliers such as medical/hospital libraries and/ or medical information centres.

19: Does your health centre have an active cooperation with the clinical library/informational centre?

- No
- Yes
- Other

20: I am familiar with the clinical information services offered in my region.

- No
- Yes

21: The last time I was in contact with a regional informational service was:

- Less than a month ago
- Less than six months
- More than a year ago

22: When was the last time you received information from your regional information service provider?

- Less than a month ago
- Less than six months
- More than a year ago.

23: Is it easy to reach out to the clinical information service?

- No
- Yes

24: Other thoughts and comments:
